# Surface Plasmon Resonance Sensors Based on the Molecularly Imprinted Technique for Simvastatin Detection

**DOI:** 10.1021/acsomega.5c07559

**Published:** 2025-12-22

**Authors:** Sona Faalnouri, Duygu Çimen, Adil Denizli, Nilay Bereli

**Affiliations:** † Bioengineering Division, 37515 Hacettepe University, Ankara 06800, Turkey; ‡ Department of Chemistry, Hacettepe University, Ankara 06800, Turkey

## Abstract

In this study, surface plasmon resonance (SPR)-based sensors were developed to determine simvastatin (SIM) with phosphate buffer (pH 7.4) using the molecular imprinting technique (MIT). SIM imprinted (MIP) and nonimprinted (NIP) poly­(2-hydroxyethyl methacrylate-*N*-methacryloyl-l-tryptophan methyl ester) polymeric films were synthesized onto the surface of the SPR chips to obtain kinetic parameters. The characterization of the MIP and NIP sensors was determined by contact angle and atomic force microscopy measurements. The range of linearity was measured as 0.001–1.0 mg/mL for SIM imprinted polymeric film-based SPR sensors. The selectivity of SPR sensors for competitive adsorption of atorvastatin and rosuvastatin was also investigated. After optimizing the experimental studies for SIM determination, SIM determination was also performed in artificial plasma solutions, and the recoveries were calculated to be approximately 99%. The findings indicate that SIM imprinted SPR sensors demonstrate exceptional sensitivity and selectivity along with a remarkably low detection limit for precise target identification.

## Graphical abstract

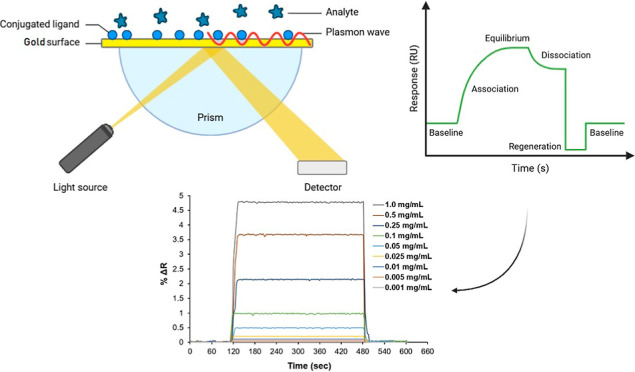

## Introduction

Simvastatin (SIM), a drug commonly used to lower cholesterol and an HMG-CoA reductase inhibitor, has been in use since 1988. When taken at the maximum recommended dosage of 80 mg per day, it generally reduces low-density lipoprotein cholesterol (LDL-C) levels by approximately 47%.^1^ SIM has attracted considerable interest for its ability to stimulate bone growth and support angiogenesis.^2–6^ Likewise, the anticancer property of SIM has been a subject of research for many years.^7–10^ So far, other related studies have also been conducted regarding the effects and characteristics of SIM.^11,12^ In this study, a polymeric film was prepared on the sensor surface by the MIP method.^13^ The molecular MIP technique involves using a functional monomer and a cross-linking agent to form a polymer matrix around the template molecule (SIM) within a solvent.^14^

The MIP technique creates specific regions in the polymer that bind to the target molecule, thereby enhancing selectivity.^15,16^ MIPs are artificially designed materials that feature selective recognition sites, which are tailored to match the shape, size, and functional groups of a particular target molecule, referred to as the template.^45,46^ These sites are created by polymerizing monomers around the template, which is later removed, leaving behind cavities that enable selective rebinding.^17,18^ MIP is widely applied for various fields including separation, drug, and chemical analysis.^19^ Recently, sensors made by molecularly imprinted polymers have gained popularity due to their exceptional selectivity and specificity.^47^ These analytical instruments produce detectable signals corresponding to varying concentrations, making them ideal for identifying biologically active substances.^20,21^ SPR sensors detect molecular binding on a metal-coated chip without needing labels.^22^ When target molecules bind to the MIP layer on the SPR sensor, they alter the resonance angle, producing a measurable signal.^23^ They operate by reflecting light between layers of differing refractive indices with binding events altering the refractive index on the sensor surface. SPR sensors offer high sensitivity, selectivity, low sample use, fast analysis, and reusability.^24–26^ While they excel in fields like diagnostics, environmental monitoring, and food safety, they do face limitations such as nonspecific binding and sterile barrier challenges.^27^ Together, MIPs and SPR offer a powerful approach for sensitive and selective detection in applications like biosensing and diagnostics.^28^ In this study, SIM imprinted (MIP) and nonimprinted (NIP) poly­(2-hydroxyethyl methacrylate-*N*-methacryloyl-l-tryptophan methyl ester) (poly­(HEMA-MATrp)) polymeric film-based SPR sensors were prepared for the determination of SIM in phosphate buffer (pH 7.4). The surface properties of MIP and NIP SPR sensors were analyzed by using atomic force microscopy (AFM) and contact angle (CA) measurements. To evaluate the interaction between SIM molecules and MIP SPR sensors, isotherm models were utilized to analyze the experimental data. Additionally, kinetic evaluations were conducted for SIM concentrations in the interval of 0.001 to 1.0 mg/mL. Selectivity assessments were carried out using MIP SPR sensors to detect SIM in aqueous solutions, which was investigated with rosuvastatin (RSV) and atorvastatin (ATV) molecules. The reusability of MIP SPR sensors was tested four times using 0.5 mg/mL SIM solution, showing no reduction in binding capacity. Finally, kinetic analyses were performed with artificial plasma solution to demonstrate the applicability of the SPR sensors.

## Experimental Section

### Materials

SIM (C_25_H_38_O_5_, ≥99%, Sigma-Aldrich, Germany) in white solid form, ethylene glycol dimethacrylate (EGDMA, 98%, Sigma-Aldrich, Germany) and azobis­(isobutyronitrile) (AIBN, 98%, Sigma-Aldrich, Germany) were purchased and used without further purification. 2-Hydroxyethyl methacrylate (HEMA, 99%, Fluka A.G., Buchs, Switzerland) was obtained from Fluka. The functional monomer *N*-methacryloyl-l-tryptophan methyl ester (MATrp), previously synthesized and reported by Denizli et al.,^29^ was employed as a suitable monomer for the SIM template molecule. Deionized water (DW) used throughout the experiments was purified using a Barnstead water purification system (Dubuque, IA, USA). SPR measurements were performed using an SPR Imager II instrument (GWC Technologies, CA, USA).

### Fabrication of MIP and NIP SPR Sensors

The modification of the gold surface of the SPR chips was applied by using allyl mercaptan (CH_2_CHCH_2_SH). The gold surface was initially immersed in an acidic piranha solution for 20 s and then thoroughly cleaned. The chips were wiped with ethanol, dried in a vacuum oven for 3 h, and subsequently coated with 5 μL of allyl mercaptan. Afterward, the chips were cleansed with ethanol and dried under a nitrogen (N_2_) atmosphere. To remove dissolved O_2_, the polymer solution was exposed to a flow of N_2_ gas for 5 min.

### Synthesis of SPR Chips

The preparation of SIM imprinted (MIP) and nonimprinted (NIP) (poly­(HEMA-MATrp)) polymeric film-based SPR sensors was carried out using the molecular imprinting technique. The prepolymerization complex solutions were prepared using different μmol ratios of MATrp monomer (30, 60, and 120 μmol) while keeping the SIM amount (1 μmol) constant. SIM:MATrp absorbance was measured with a UV–vis spectrophotometer (SHIMADZU UV-1601 model, Tokyo, Japan) in the wavelength range 200–700 nm. The highest absorbance value for the synthesis of the SIM imprinted polymeric film was obtained in the SIM:MATrp prepolymerization solution at a μmol ratio of 1:120 (Figure 1).

**Figure 1:**
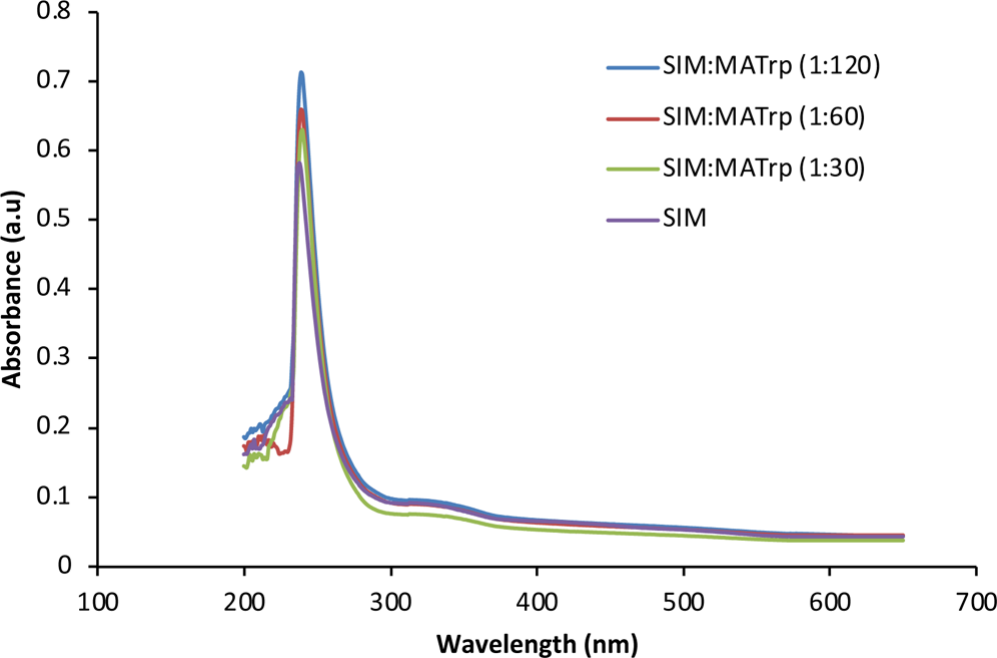
UV spectrum of the SIM:MATrp prepolymerization complex.

In molecular imprinting, to ensure the creation of selective binding sites on the sensor surface, the prepolymerization complex consisting of SIM and MATrp was prepared by mixing SIM:MATrp (1:120 μmol ratio) for 3 h. To create the prepolymerization complex, 0.5 mg of SIM was first dissolved in water, followed by the addition of 60 μL of MATrp. This process helps to form precise molecular cavities for selective binding during the polymerization stage. Then, the SIM imprinted poly­(HEMA-MATrp) MIP SPR sensors were synthesized by polymerizing the (SIM:MATrp) complex with HEMA (50 μL) and EGDMA (100 μL). Following the addition of 4 mg of AIBN as an initiator, a 4 μL solution was applied to the chip surface. This process resulted in gold-coated SPR chips with covalently attached allyl groups. The NIP SPR sensors were produced without the inclusion of a template molecule (SIM) in their preparation. NIP (nonimprinted polymer) is used in the same procedure as the preparation of the MIP SPR sensor; the only difference is that the SIM molecule is not used. All other monomers, cross-linkers, and initiator amounts and polymerization conditions are the same. The prepared chip underwent photopolymerization under a UV lamp for 30 min, enabling the formation of a cross-linked polymer network on the chip surface. This process involved the activation of photoinitiators, which facilitated the polymerization of monomers in the solution. After polymerization, ethyl alcohol was used as a washing agent to remove any unreacted monomers or loosely bound components, ensuring a clean, functionalized surface. After each kinetic analysis, SIM imprinted SPR chip surfaces are washed in the SPR device for 1 h with a 0.1 M NaCl desorption solution. The removal of SIM molecules from the SPR sensor surface was periodically monitored both in the SPR device and by using UV–vis spectroscopy. After this process, before starting a new kinetic analysis, SIM imprinted SPR sensor surfaces are first equilibrated for 30 min with deionized water and PBS buffer (pH 7.4). After these processes, kinetic analysis is performed using SIM imprinted SPR sensor surfaces with SIM solutions prepared at different concentrations in adsorption experiments. A schematic of these preparation steps is presented in Figure 2.

**Figure 2:**
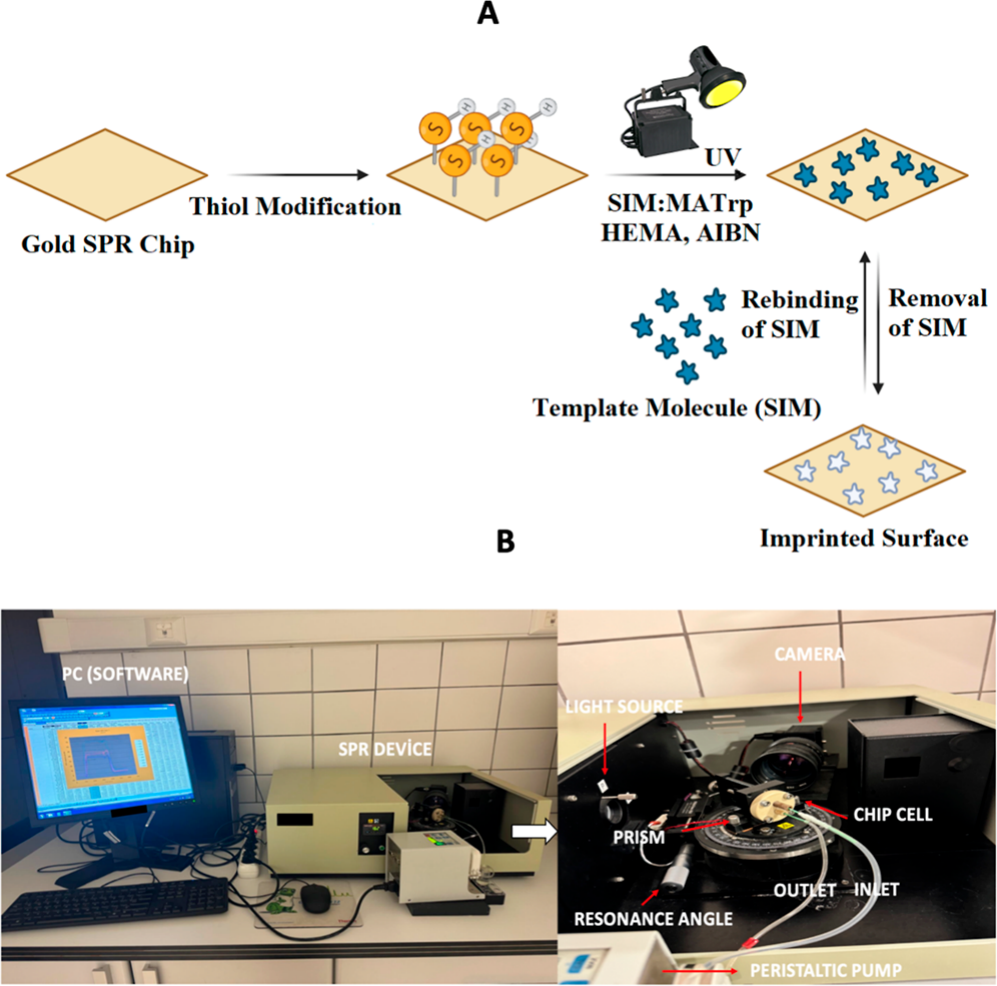
(A) Process for preparing the SIM imprinted SPR chip surface and the (B) SPR Imager II system.

### Characterization of SPR Sensor Surfaces

The depth, thickness, and hydrophilicity of both SIM imprinted poly­(HEMA-MATrp) (MIP) and nonimprinted poly­(HEMA-MATrp) (NIP) SPR surface were evaluated using different characterization techniques, such as atomic force microscopy (AFM) and contact angle analysis. A drop shape analyzer system (Kruss DSA 100, Germany) was employed to evaluate the wettability and hydrophilicity of the sensor surfaces. Surface morphology analysis was conducted using AFM (Nanomagnetics Instruments, Oxford, UK) in tapping mode at a scanning speed of 1 μm/s. The analysis provided a resolution of 256 × 256 pixels over a 1000 nm × 1000 nm area.^30,31^

### Real-Time Kinetic Analyses

The kinetic analysis for detecting SIM was conducted using the SPR Imager II (GWC Technologies, WI, USA), employing phosphate-buffered saline (PBS) as the medium. Throughout the kinetic analysis, a flow rate of 150 μL/min (0.031″ ID tubing) was maintained, with an operating wavelength of 800 nm and a prism made of SF10 glass. A sample volume of 2 mL was utilized for the equilibration, adsorption, and desorption phases.

SIM solutions were prepared within concentrations in the interval of 0.001 to 1.0 mg/mL. Each analysis involved equilibrating SIM imprinted SPR sensors by passing PBS buffer (pH 7.4) through the system at a flow rate of 150 μL/min for 2 min. SIM solutions at different concentrations were added into the SPR system for 6 min for kinetic analysis. As a final step, a 0.1 M NaCl solution was passed through the SPR sensor surface for 2 min as a desorption solution to remove SIM molecules bound to the SPR sensor surface. In kinetic analyses, the cycles of equilibrium, adsorption, and desorption were made in 10 min and the refractive index changes (% Δ*R*) were realized in real-time measurements.

Adsorption isotherm models Langmuir, Freundlich, and Langmuir–Freundlich isotherm models were calculated using the data obtained from kinetic analyses with SIM solutions prepared at different concentrations. The interaction between SIM molecules and the SIM imprinted SPR sensor was studied by three adsorption isotherms:

1 $$\Delta R=\left(\Delta R_{\max}C/K_{D}+C\right)Langmuir$$

2 $$\Delta R=\left(\Delta R_{\max}C^{1/n}\right)Freundlich$$

3 $$\Delta R=\left(\Delta R_{\max}C^{1/n}/K_{D}\right)Langmuir-Freundlich$$

By measuring the signal induced by SIM molecules bonding, the SPR sensor system’s response is measured as Δ*R*. The concentration of SIM is *C*, which is commonly expressed as mg/mL. The equilibrium constants for association and dissociation are *K*_A_ (mg/mL)^−1^ and *K*_D_ (mg/mL). 1/*n* represents the Freundlich constant.^32^

In this study, the SPR sensor surface was altered to form highly specific and selective molecular cavities intended to identify the target molecule, which is one of the main benefits of molecular imprinting. This approach combined the sensitivity of SPR sensors with the selectivity of molecular imprinting techniques, enabling precise molecular recognition. Both MIP and NIP SPR sensors were fabricated to assess their performance and selectivity. These customized surfaces provide an advanced platform for targeted detection and molecular analysis. To assess the selectivity of the SPR sensors, rosuvastatin (RSV, C_22_H_28_FN_3_O_6_S, *M*_W_: 481.539 g/mol) and atorvastatin (ATV, C_33_H_35_FN_2_O_5_, *M*_W_: 558.64 g/mol) were chosen as competitor molecules, which are structurally and molecularly similar to SIM (SIM, C_25_H_38_O_5_, *M*_W_: 418.56 g/mol). Selectivity experiments were performed using both MIP and NIP SPR sensors, where solutions of each competitor molecule were prepared at a concentration of 0.5 mg/mL. The selectivity coefficient (*k*) and relative selectivity coefficient (*k*’) were calculated from the data obtained during the selectivity analysis using the following equations.

4 $$k\;\left(selectivity\;coefficient\right):\Delta R_{template}/\Delta R_{competitor}$$

5 $$k^{\prime }\left(relative\;selectivity\;coefficients\right):k_{MIP}/k_{NIP}$$

6 $$\mathrm{IF}\left(Imprinting\;factor\right):\Delta R_{MIP}/\Delta R_{NIP}$$

The desorption ensured that the sensors were reusable, enhancing the reliability and repeatability of the process. These steps were critical for generating accurate kinetic data for SIM detection, providing insights into binding dynamics and affinity. The reusability of the SIM imprinted SPR sensors was thoroughly investigated by performing four successive adsorption–desorption–regeneration cycles on the same sensor chip using a 0.5 mg/mL SIM solution. For each cycle, kinetic analyses were conducted to assess the performance.

After each adsorption phase was completed, desorption was carried out using 0.1 M NaCl as the desorption agent, effectively regenerating the sensor surface. In addition, the kinetic analysis of the SIM imprinted SPR sensor was performed at various intervals such as first, second, fourth, and sixth months at 0.5 mg/mL SIM concentration, and the shelf life and reusability of the prepared sensor were investigated.

### SIM Detection from Artificial Plasma

After kinetic analyses with different solutions prepared with SIM solutions prepared in pH 7.4 phosphate buffer, the detection of SIM was also performed in artificial plasma solutions. For this purpose, solutions containing 0.25, 0.5, and 1.0 mg/mL SIM in artificial plasma solutions were prepared. First, the SPR system was equilibrated by adding pH 7.4 phosphate buffer to the system for 2 min. Then, the prepared artificial plasma solutions were introduced into the SPR system separately for 6 min. As a final step, the desorption solution, 0.1 M NaCl, was passed for 2 min, and the obtained SPR sensorgrams were recorded in real time.

## Results and Discussion

### Characterization Studies of SPR Sensor Surfaces

In this study, contact angle analysis was used to measure the wettability of a surface with a liquid. It quantified the angle formed between the surface and the tangent of a liquid droplet at the point of contact. The contact angle measurements for the unmodified SPR chip, SIM imprinted poly­(HEMA-MATrp) (MIP), and nonimprinted poly­(HEMA-MATrp) (NIP) SPR surface were found to be 79.1° (Figure 3A), 81.3° (Figure 3B), and 69.8° (Figure 3C), respectively. Figure 1B introduces the nonimprinted poly­(HEMA-MATrp) (NIP) SPR sensor surface, including the hydrophobic groups (such as aromatic rings) of the functional monomer (MATrp), which make the surface more hydrophobic, leading to a higher contact angle with water. On the contrary, the observed reduction in contact angles of the SIM imprinted poly­(HEMA-MATrp) (MIP) SPR sensor surface in Figure 3C indicates an increase in surface hydrophilicity, aligning with the hydrophilic properties of the SIM molecule used in this study. This indicates that water spreads out more on the surface, meaning that the surface has a strong affinity for water. The lower the contact angle, the more hydrophilic the surface is.^33^ Additionally, atomic force microscopy (AFM) was used to measure the forces between the sharp probe and the SPR sensor surfaces to characterize surface morphology, roughness, mechanical properties, and other surface characteristics of SIM imprinted poly­(HEMA-MATrp) (MIP) and nonimprinted poly­(HEMA-MATrp) (NIP) surface at the atomic or molecular level.^34^ So, the deepness of polymeric films was determined with AFM images in Figure 4. The surface depth values of MIP and NIP SPR sensors were determined as 86.11 nm vs 82.49 nm, respectively. According to the AFM results, SPR sensor surfaces clearly shows that a polymeric film was successfully synthesized onto the SPR sensor surfaces.

**Figure 3:**
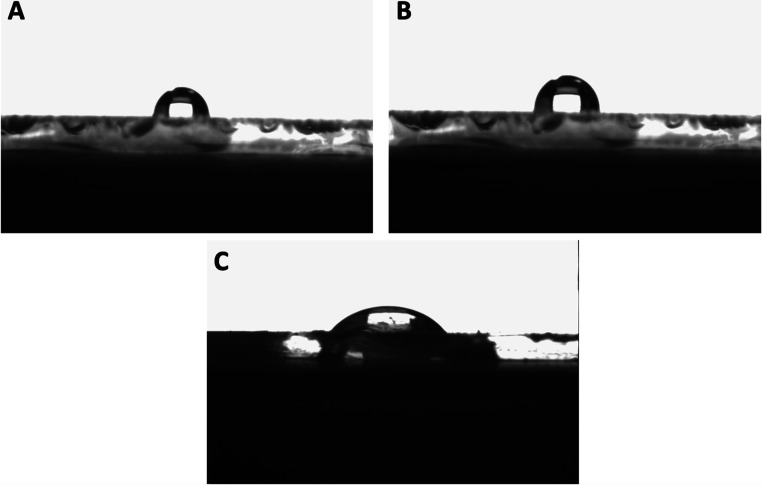
Morphology of SPR sensor surfaces: contact angles of (A) unmodified SPR chip surface, (B) SIM imprinted poly­(HEMA-MATrp) (MIP), and (C) nonimprinted poly­(HEMA-MATrp) (NIP) SPR surface.

**Figure 4:**
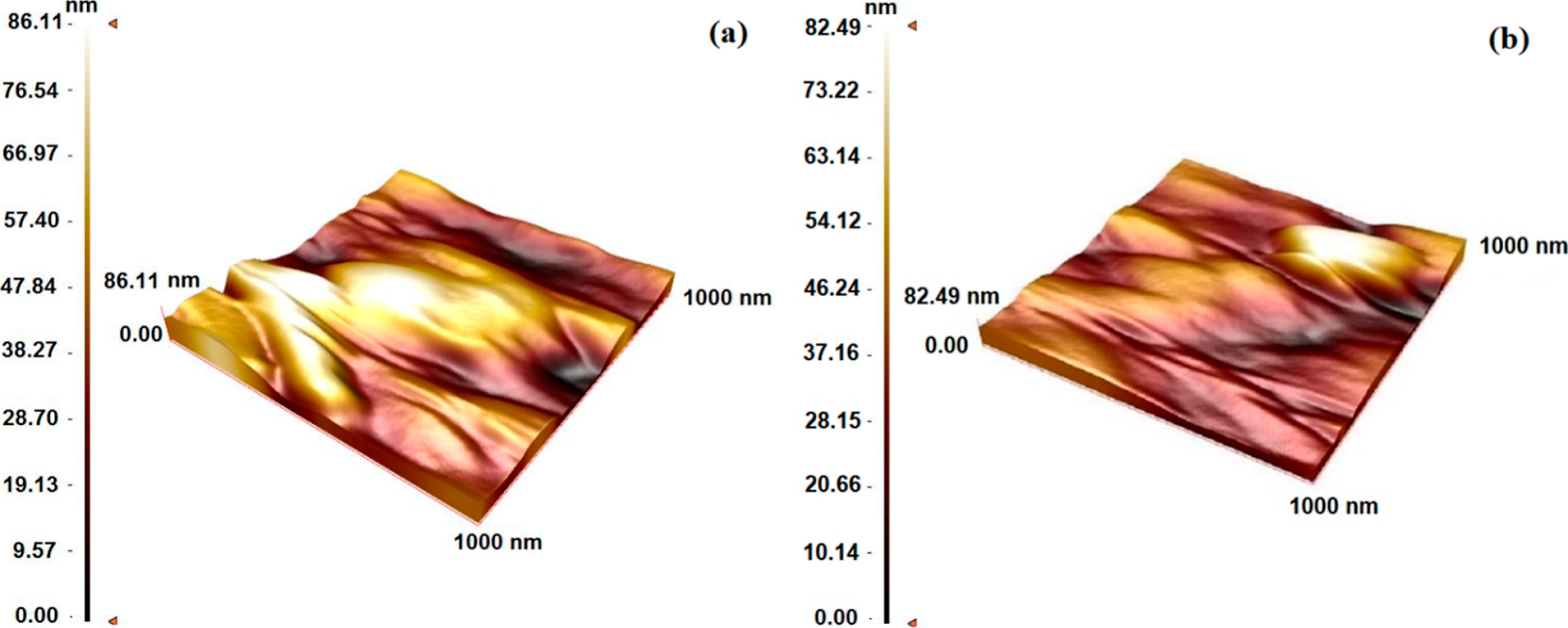
Morphology of SPR sensor surfaces. AFM studies: (A) SIM imprinted poly­(HEMA-MATrp) (MIP) and (B) nonimprinted poly­(HEMA-MATrp) (NIP) surface.

### Kinetic Analysis

Kinetic studies for the determination of SIM were carried out with SIM imprinted poly­(HEMA-MATrp) (MIP) and nonimprinted poly­(HEMA-MATrp) (NIP) SPR surface. As known, medium pH affects the complexation reaction between SIM and functional monomer (MATrp); the ideal pH was identified as pH 7.4 phosphate buffer for detecting SIM. To evaluate the correlation between the SPR signal and SIM concentration, SIM solutions concentrations in the interval of 0.001 to 1.0 mg/mL were prepared. The SPR sensors interacted with these solutions, and the kinetic data were analyzed using SPRview software. Sensorgrams obtained from SIM solutions at various concentrations and the graphs of refractive index versus time for different SIM concentrations applied to the sensors are shown in Figure 5a,b. The calibration graph of the SPR sensor with SIM concentrations in the range of 0.001–1.0 mg/mL is presented in Figure 5c. The % Δ*R* values obtained for the SIM imprinted poly­(HEMA-MATrp) MIP sensor were higher compared to those of the nonimprinted poly­(HEMA-MATrp) NIP sensor. The results revealed the existence of molecular cavities specifically designed for the binding of SIM molecules.

**Figure 5:**
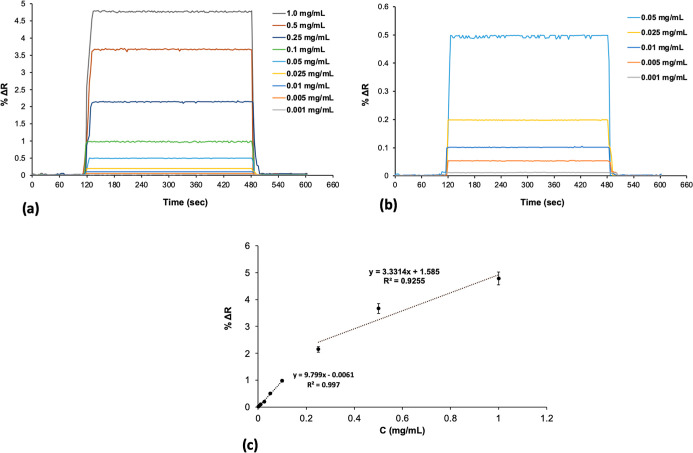
Kinetic analysis. SIM concentration range of (a) between 0.001 and 1.0 mg/mL and (b) between 0.001 mg/mL and 0.05 mg/mL and the calibration graph of (c) SIM imprinted SPR sensor aqueous solutions of SIM at different concentrations (*n*: 3).

As the SIM concentration increases, the rise in refractive index values can be attributed to the increasing concentration gradient, which promotes the molecular interaction between the solution and the surface. Before each measurement, the SPR system was equilibrated with a phosphate buffer (pH 7.4), and solutions prepared at various SIM concentrations were given to the SPR system. After each analysis, SPR sensors were regenerated by washing with 0.1 M NaCl and phosphate buffer, completing the equilibrium adsorption–desorption cycle in approximately 8 min. The equations (*y* = 9.799*x* – 0.0061) and (*y* = 3.3314*x* + 1.585) were derived from the concentration-Δ*R* graphs corresponding to the low (0.001 mg/mL) and high (1.0 mg/mL) SIM concentration ranges, respectively. The linearity coefficients were found to be (*R* ^2^ = 0.997) and (*R* ^2^ = 0.9255), respectively, indicating 99% accuracy in the measurements.

Based on the kinetic data, the limit of detection (LOD) and limit of quantification (LOQ) values for MIP and NIP SPR sensors were calculated as 0.00015 and 0.00051 mg/mL, respectively. Limit of detection (LOD) and limit of quantification (LOQ) values were calculated with the equations:

7 LOD=3.3S/m

8 LOQ=10S/m

where “*S*” is the standard deviation of the intercept and “*m*” is the slope of the regression line.^35^

Isotherm models were used for extracting meaningful information from SPR data, enabling precise and accurate interpretation of molecular interactions on surfaces.^36^ Therefore, the Freundlich, Langmuir, and Langmuir–Freundlich isotherm models were used to examine the interactions between SIM and the MIP SPR sensor, as shown in Figure 6. When analyzing the correlation coefficient (*R* ^2^) values from Table 1, tt was observed that the Langmuir isotherm model provided the best fit for the interactions between SIM molecules and the MIP SPR sensor. The linearity of the Langmuir model was better than that of the Freundlich and Langmuir–Freundlich models. The Δ*R*_max_ value calculated from the Langmuir isotherm model was very close to the experimentally obtained value. The highest signal value obtained from the Langmuir isotherm was Δ*R*_max_: 2.096. These findings indicate that the binding properties of SIM on the MIP SPR sensor surface are homogeneous and single-layered and exhibit low lateral interactions.

**Figure 6:**
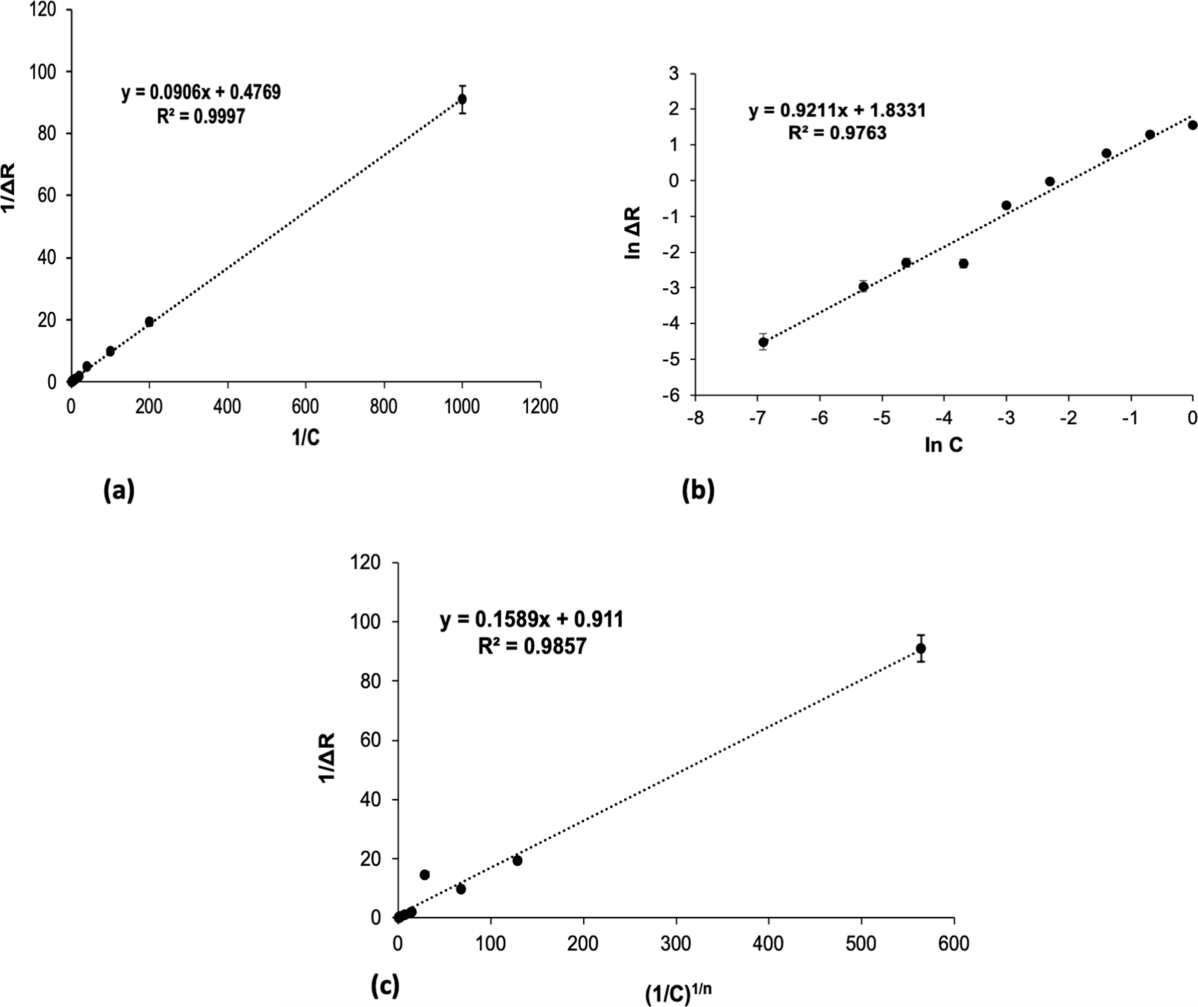
Isotherm models: Langmuir (a), Freundlich (b), and Langmuir–Freundlich (c).

**Table 1:** Isotherm Parameters for SIM Imprinted SPR Sensors

Langmuir	Freundlich	Langmuir–Freundlich
Δ*R*_max_: 2.096 mg/mL	Δ*R*_max_: 6.253 mg/mL	Δ*R*_max_: 1.097 mg/mL
*K*_D_: 0.189 mg/mL	1/*n*: 0.9211	1/*n*: 0.9211
*K*_A_: 5.263 (mg/mL^)–1^	*R* ^2^: 0.976	*K*_D_: 0.174 mg/mL
*R* ^2^: 0.999		*K*_A_: 5.73 (mg/mL)^−1^
		*R* ^2^: 0.985

The Langmuir model exhibited a higher correlation coefficient (*R* ^2^ = 0.999) compared to the Freundlich model (*R* ^2^ = 0.9763), indicating that the adsorption of SIM molecules onto the MIP SPR surface occurs primarily as a monolayer on a homogeneous surface. This suggests that the imprinted cavities are uniform and specific to SIM molecules, confirming the successful molecular imprinting process.

### Selectivity Studies

The selectivity of SIM imprinted SPR sensors was compared with kinetic analyses performed with nonimprinted SPR sensors. Kinetic analysis of the NIP SPR sensors was performed using SIM solution at 0.5 mg/mL. The selectivity of the MIP SPR sensor was investigated with rosuvastatin (RSV) and atorvastatin (ATV) molecules. The selective molecules were selected based on their structural and molecular weight similarities to SIM. Selective recognition of SIM with MIP SPR sensor was investigated using 0.5 mg/mL of SIM (C_25_H_38_O_5_, *M*_W_: 418,56 g/mol), rosuvastatin (RSV, C_22_H_28_FN_3_O_6_S, *M*_W_: 481,539 g/mol), and atorvastatin (ATV, C_33_H_35_FN_2_O_5_, *M*_W_: 558,64 g/mol) molecules (Figure 7). The NIP SPR sensor demonstrates a minimal ability to detect SIM and other competitor molecules. The relative selectivity of the MIP and NIP SPR sensors is illustrated in Figure 8a,b. Based on the experimental results, the selectivity coefficients (*k*) and relative selectivity coefficients (*k*’) for simvastatin (SIM), rosuvastatin (RSV), and atorvastatin (ATV) were calculated for both MIP and NIP SPR sensors, with comparisons made between RSV and ATV molecules relative to SIM (Table 2).

**Figure 7:**
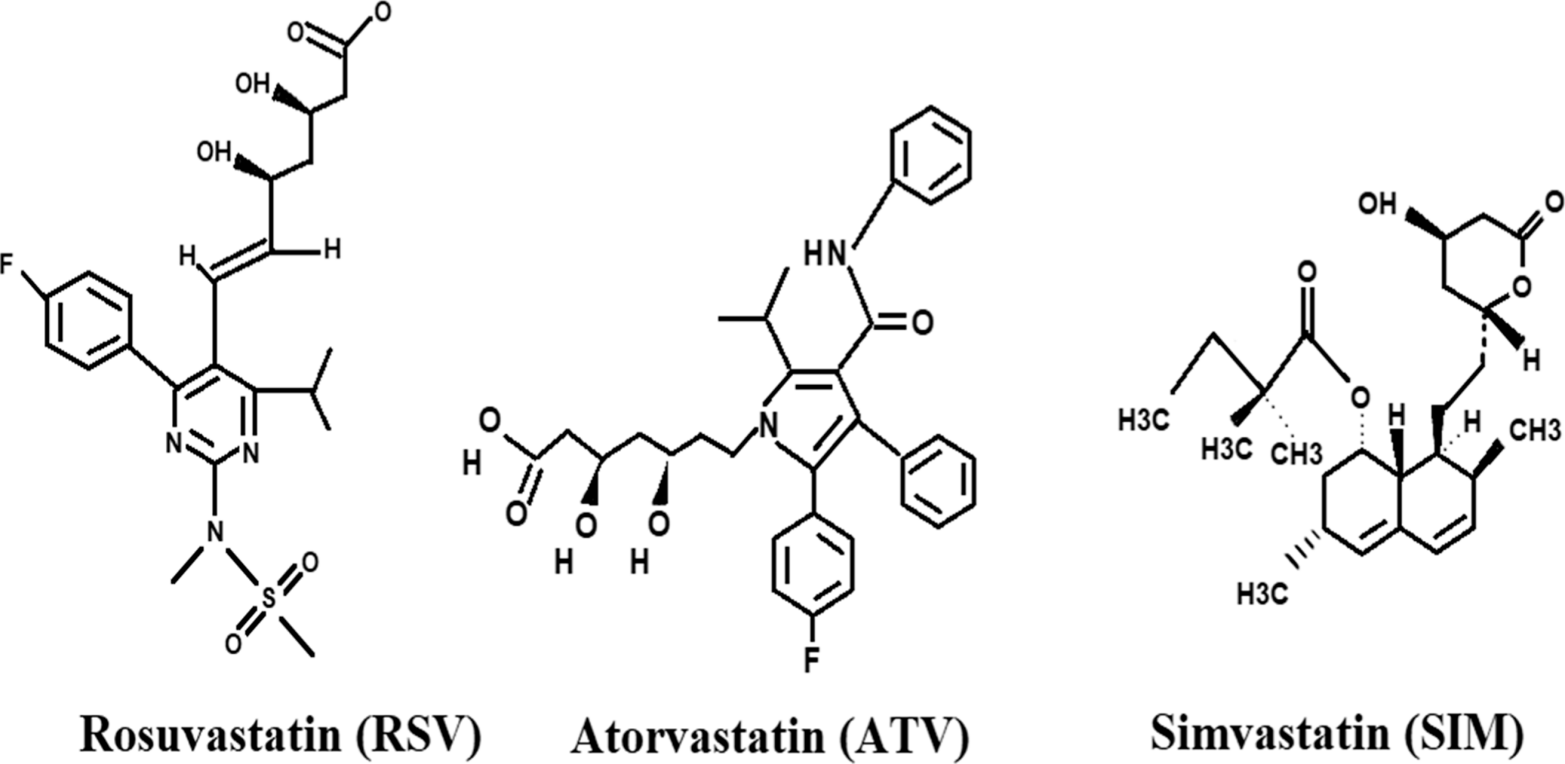
Chemical structure of rosuvastatin (RSV), atorvastatin (ATV), and simvastatin (SIM) molecules used in the selectivity studies of SPR sensors.

**Figure 8:**
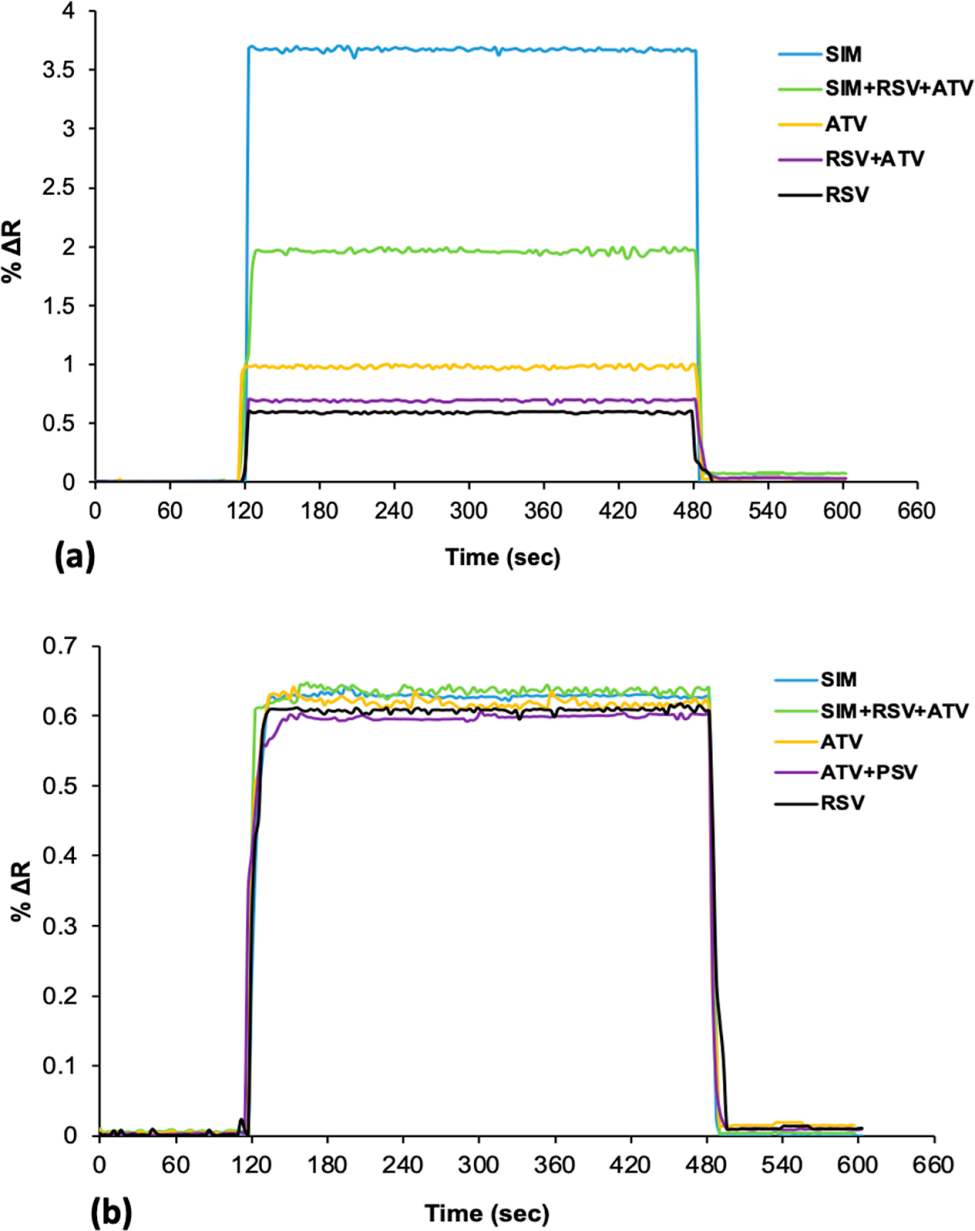
Selectivity studies for MIP (a) and NIP (b) SPR sensors (*n*: 3).

**Table 2:** Selectivity and Relative Selectivity Coefficients of MIP and NIP SPR Sensors for Competitive Molecules

	MIP SPR sensor		NIP SPR sensor		
biomolecules	% Δ*R*	*k*	% Δ*R*	*k*	*k*’
SIM	3.655	–	0.624	–	–
ATV	0.974	3.752	0.595	1.048	3.580
RSV	0.597	6.122	0.609	1.024	5.978
ATV + RSV	0.698	5.236	0.628	0.993	5.272
SIM + ATV + RSV	1.950	1.874	0.642	0.971	1.929

### Reusability and Stability

One significant advantage of MIPs is their reusability and stability. The reusability of SIM imprinted poly­(HEMA-MATrp) SPR sensors was tested four times using 0.5 mg/mL SIM solution, showing no reduction in binding capacity. Stability tests revealed that these sensors maintained their performance under long-term storage conditions, confirming their durability and reliability for repeated use. Initially, MIP SPR sensors were equilibrated using phosphate buffer (pH 7.4) for 2 min. Following this, a SIM solution at a concentration of 0.5 mg/mL was applied to the SPR system for 6 min. To remove the bound SIM molecules from the SPR sensor surface, MIP SPR sensors were treated with a 0.1 M NaCl solution for 2 min. The reusability performance of the SIM imprinted SPR sensors is presented in Figure 9a. The efficiency of the SPR sensors was determined to be 98%.

**Figure 9:**
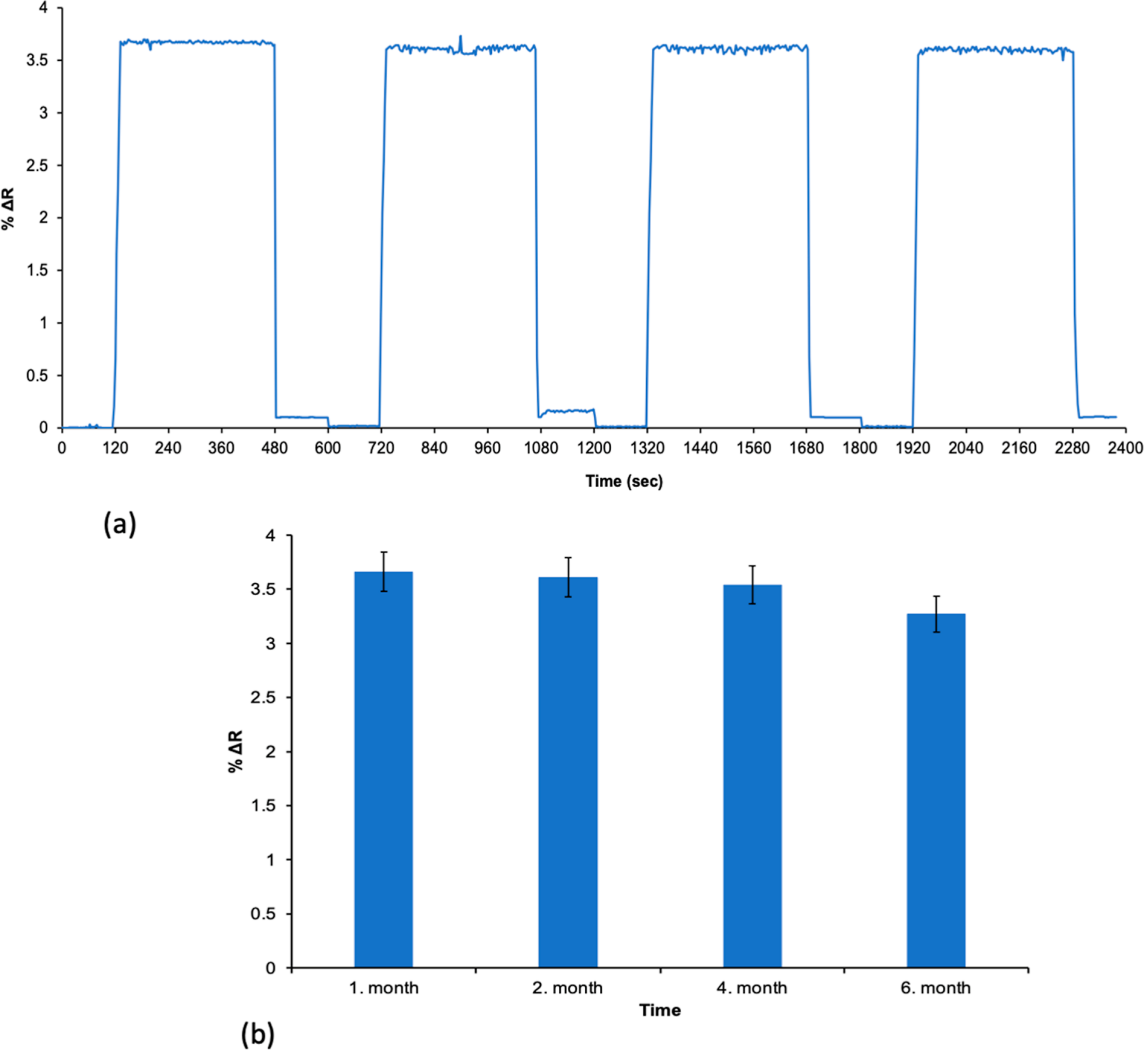
Reusability of MIP SPR sensors: (a) short-term and (b) long-term stability) (*n*: 3).

The efficiency and stability of the MIP SPR sensor were investigated by kinetic analyses performed with 0.5 mg/mL SIM aqueous solution at different months (1 month, 2 months, 4 months, and 6 months). Sensorgrams of kinetic analyses for the determination of the SIM molecule at different times are given in Figure 9b. As a result of kinetic analyses performed under the same conditions and using the same chip, it was observed that the performance of the MIP SPR sensor decreased by 10.66% after 6 months (Figure 9b). This result shows that there is no significant decrease in the performance of the MIP SPR sensor and that it can be used repeatedly.

### Detection of SIM in Artificial Plasma Samples

Kinetic analyses for the determination of SIM in artificial plasma solutions were investigated using MIP SPR sensors. For this purpose, artificial plasma solutions containing 0.25, 0.5, and 1.0 mg/mL SIM were prepared. Kinetic analysis was performed on artificial plasma solutions prepared separately with concentrations of 0.25, 0.5, and 1.0 mg/mL. First, pH 7.4 phosphate buffer was given into the SPR system for 2 min. After that, the spiked artificial plasma solutions with SIM solution concentrations of 0.25, 0.5, and 1.0 mg/mL were given into the SPR system for 6 min. Finally, 0.1 M NaCl solution was passed through the SPR system as a desorption solution for 2 min, and the real-time kinetic analyses were performed (Figure 10). For the determination of SIM, the recoveries obtained at 0.25, 0.5, and 1.0 mg/mL SIM concentrations spiked to artificial plasma solutions were calculated to be 99.93%, 99.68%, and 100.15%, respectively. The LOD value of SIM in artificial plasma was calculated to be 0.00029 mg/mL.

**Figure 10:**
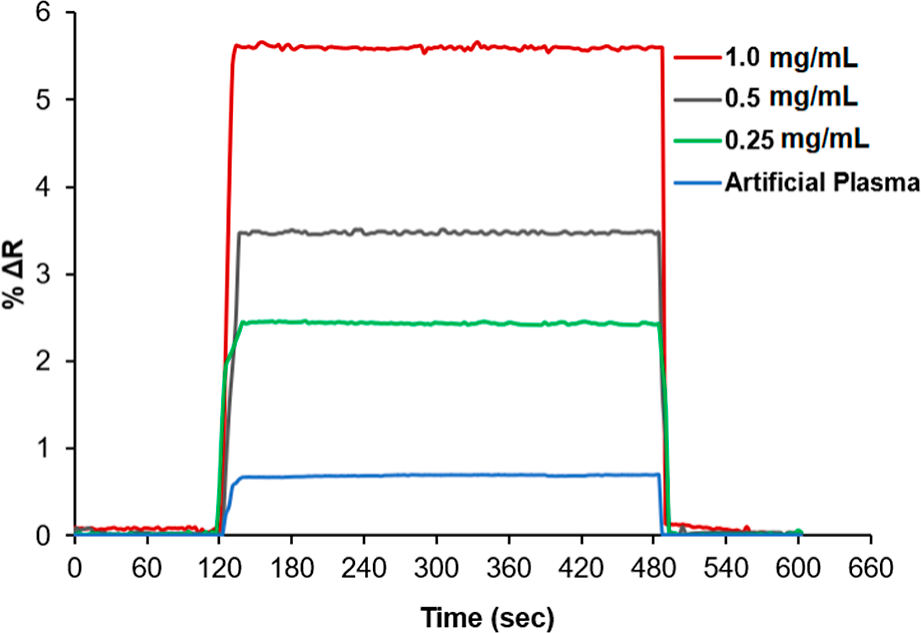
SPR sensorgram for the determination of SIM in artificial plasma solutions with concentrations in the range of 0.25–1.0 mg/mL (*n*: 3).

In the present study, using molecular imprinting technology, MIP polymeric films were synthesized onto the surface of SPR chips to obtain kinetic parameters. Here is a comparative summary of the limit of detection (LOD) for SIM across various validated analytical methods reported in previous studies (Table 3).

**Table 3:** Comparative Overview of the Limit of Detection (LOD) for SIM

analytical method	detection technique	LOD (μg/mL or ng/mL)	sample matrix	reference
HPLC	UV detection	0.02 μg/mL	bulk and tablet	^37^
HPLC	diode array detector (DAD)	0.01 μg/mL	plasma	^38^
RP-HPLC	UV detection	0.03 μg/mL	pharmaceutical dosage	^39^
LC–MS/MS	mass spectrometry	0.2 ng/mL	human plasma	^40^
UPLC	photodiode array detector	0.015 μg/mL	bulk	^41^
spectrofluorimetry	fluorescence	0.01 μg/mL	tablet formulation	^42^
UV–vis spectrophotometry	UV detection	0.05 μg/mL	bulk and tablet	^43^
electrochemical (voltammetry)	differential pulse voltammetry	0.005 μg/mL	pharmaceutical formulation	^44^

## Conclusion

A variety of analytical techniques have been reported for the quantification of pharmaceutical compounds across different matrices, each offering a distinct sensitivity and applicability. Among chromatographic methods, HPLC coupled with UV detection has shown good sensitivity with a detection limit of 0.02 μg/mL in bulk and tablet formulations, while diode array detection (DAD) enhances the sensitivity further to 0.01 μg/mL in plasma samples. Reverse-phase HPLC (RP-HPLC) with UV detection demonstrates slightly higher detection limits (0.03 μg/mL) but remains widely applicable to pharmaceutical dosage forms. Advanced hyphenated techniques such as LC–MS/MS achieve superior sensitivity, detecting as low as 0.2 ng/mL in human plasma, making them ideal for bioanalytical studies. UPLC with photodiode array detection also offers excellent sensitivity (0.015 μg/mL) in bulk analysis, reflecting improvements in resolution and speed compared with conventional HPLC. Among spectroscopic approaches, spectrofluorimetry achieves a detection limit of 0.01 μg/mL in tablet formulations, outperforming conventional UV–vis spectrophotometry (0.05 μg/mL) in sensitivity. Notably, electrochemical methods such as differential pulse voltammetry exhibit the lowest detection limit (0.005 μg/mL), highlighting their potential for highly sensitive determination in pharmaceutical formulations. Overall, while chromatographic and mass spectrometric techniques offer broad applicability and superior sensitivity for complex matrices like plasma, spectroscopic and electrochemical methods provide simpler, cost-effective alternatives for routine quality control in bulk and dosage forms.

To examine the binding kinetic studies of SIM, MIP and NIP SPR sensors made of poly­(HEMA-MATrp) were prepared. Kinetic analyses were performed by preparing SIM solutions at different concentrations, and the lowest detection limit was 0.00015 mg/mL. Using the obtained kinetic data, different isotherm models were examined, revealing the best fit between the SPR sensor and SIM molecules with the Langmuir isotherm model. This suggests that the SIM binding properties on the SPR sensor surface are homogeneous and monolayered and have low lateral interactions. The binding equilibrium constant (*K*_A_: 5.263 (mg/mL)^−1^) being higher than the dissociation equilibrium constant (*K*_D_: 0.189 mg/mL) indicates a high affinity of the SIM molecule for the SIM imprinted SPR sensor. When the shelf life and performance of the SPR sensor were examined on the same day and at different times, a decrease of approximately 10.66% was observed in different months, and a decrease of 2.45% was observed within the same day. These results show us that the shelf life and performance of the SPR sensors are less after use, thanks to the stable structures of the polymers on the SPR sensor surfaces prepared by molecular imprinted technology. Besides, the competitive analysis showed that SIM has a higher tendency to bind to the phosphate buffer (pH 7.4).
